# Volatile Methyl Siloxanes and Other Organosilicon Compounds in Residential Air

**DOI:** 10.1021/acs.est.2c05438

**Published:** 2022-11-03

**Authors:** Betty Molinier, Caleb Arata, Erin F. Katz, David M. Lunderberg, Yingjun Liu, Pawel K. Misztal, William W Nazaroff, Allen H. Goldstein

**Affiliations:** † Department of Civil and Environmental Engineering, University of California, Berkeley, California 94720, United States; ‡ Department of Chemistry, University of California, Berkeley, California 94720, United States; § Department of Environmental Science, Policy and Management, University of California, Berkeley, California 94720, United States; ∥ College of Environmental Sciences and Engineering, Peking University, Beijing 100871, China; ⊥ Civil, Architectural, and Environmental Engineering, The University of Texas at Austin, Austin, Texas 78712, United States

**Keywords:** cyclic volatile methyl siloxane, linear volatile methyl siloxane, emissions, source attribution, indoor air

## Abstract

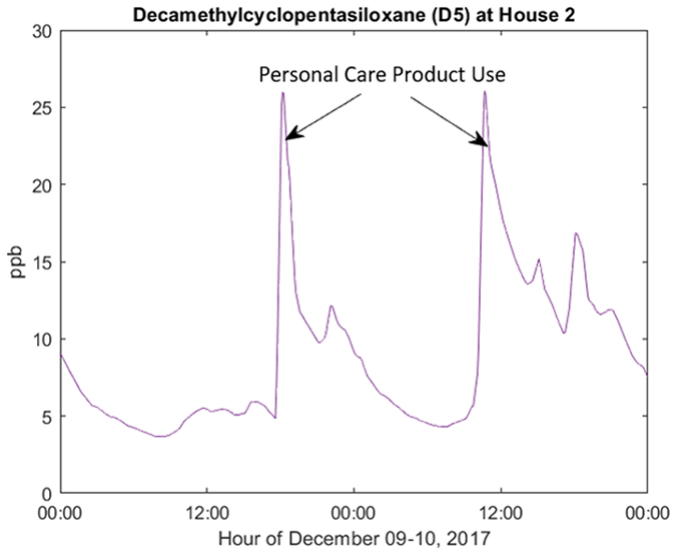

Volatile methyl siloxanes (VMS) are ubiquitous in indoor environments due to their use in personal care products. This paper builds on previous work identifying sources of VMS by synthesizing time-resolved proton-transfer reaction time-of-flight mass spectrometer VMS concentration measurements from four multiweek indoor air campaigns to elucidate emission sources and removal processes. Temporal patterns of VMS emissions display both continuous and episodic behavior, with the relative importance varying among species. We find that the cyclic siloxane D5 is consistently the most abundant VMS species, mainly attributable to personal care product use. Two other cyclic siloxanes, D3 and D4, are emitted from oven and personal care product use, with continuous sources also apparent. Two linear siloxanes, L4 and L5, are also emitted from personal care product use, with apparent additional continuous sources. We report measurements for three other organosilicon compounds found in personal care products. The primary air removal pathway of the species examined in this paper is ventilation to the outdoors, which has implications for atmospheric chemistry. The net removal rate is slower for linear siloxanes, which persist for days indoors after episodic release events. This work highlights the diversity in sources of organosilicon species and their persistence indoors.

## Abstract 2

This study reports time-resolved and average concentrations, emission rates, and source apportionment of several organosilicon species in residential indoor environments.

## Introduction

Organosilicon species possess one or more C–Si bonds. These compounds can assume various configurations (such as pyramidal, cyclic, or folded), undergo rearrangements, and sometimes exhibit hypervalency due to the stability of the silicon atom resulting from an unpaired valence electron in the low-energy d-orbital.^1,2^ Physicochemical properties vary widely by subgroup, among which are siloxanes, silanols, orthosilicates, silanes, and silazanes.^3^ Organosilicon compounds have industrial and medical applications, such as silicone breast implants, and are also found in many consumer products, such as cosmetics and cooking materials.^3,4^

Volatile methyl siloxanes (VMS) comprise a subgroup of organosilicon compounds, possessing rings (cyclic, “D”) or chains (linear, “L”) of alternating silicon and oxygen atoms with attached methyl groups (−CH_3_).^5^ As these structures lead to some differences in behavior despite similar molecular weights,^6^ cyclic and linear VMS are examined separately in this paper. They volatilize easily despite their high molecular weights, and, because of their low surface tension, high stability, and smooth texture,^5,7−9^ they are commonly used in personal care products, such as antiperspirants and deodorants, lotions, and hair care products. Laboratory studies have characterized siloxane emissions from three-dimensional (3D) printers, baking molds, and nanofilm spray.^10−12^ They are also components of siliconized rubber materials, industrial cleaning products, lubricants, and polymer formulations such as polydimethylsiloxane (PDMS).^5,7,8^ Many commercial products list blends of siloxanes, using terms such as “cyclomethicone” or “cyclosiloxanes,” rather than reporting the individual siloxane species, making it difficult to identify the actual composition or to determine potential emissions. Species also can be present as impurities rather than as a component of the intended formulation.^5^

Several studies have modeled the distribution and behavior of siloxanes in the atmosphere.^13−16^ Vapor pressure and environmental partition coefficients have been determined at various temperatures.^6,17,18^ Outdoors, oxidation of VMS initiated by the OH radical is moderately fast (lifetime ∼ 5 days, assuming an OH concentration of 10^6^ molecules/cm^3^). Indoors, the lower OH concentrations and short residence times (hours or less) mean that siloxanes are effectively inert. Therefore, the main removal process of volatilized VMS from indoor environments is ventilation, potentially modulated by reversible surface uptake.^19−23^ In the natural environment, siloxane species are bioaccumulative and have been found in human breast milk as well as in the blood and tissue of humans and various marine animals.^8,24−26^ Both D4 and D5 have been classified as endocrine and reproductive disruptors.^7^ Although evidence about health risks associated with VMS exposure remains inconclusive,^27^ some European countries have set guidelines for VMS concentrations in indoor air.^28^

Several studies have reported time-averaged levels of different VMS species in various indoor environments, such as museums, commercial buildings, and call centers^29−31^ with some attention directed at inhalation or dermal exposure to these compounds.^7,8,32−34^ The cyclic VMS D5 was found to be the most abundant of the many measured volatile organic compounds (VOCs) emitted from students in a university classroom compared to metabolically generated VOCs, such as acetone and isoprene.^35,36^ The behavior and dynamics of VMS species other than D5^37^ have not been widely studied. Prior to the university classroom study,^35^ little information was available about the temporal dynamics of VMS indoors. Likely due to the variability in personal care product usage across households and in different countries, reported average levels of siloxanes indoors vary widely. Also noteworthy is the experimental finding from Wang et al.:^38^ although D5 siloxane is volatile under atmospheric conditions, it can behave like a semivolatile compound indoors. Reversible partitioning of siloxane species to indoor surfaces would affect the time pattern of concentrations and associated exposures.

This paper presents information about VMS emission sources and removal rates that control temporal dynamics in residential air. We also present information on indoor air concentrations of three additional silicon-containing compounds: caprylyl methicone (CM),^39^ silyl acetate (SA), and C_7_H_20_O_3_Si_3_ (C7).^39^ The data originate from intensive, time-resolved, in situ measurements at three field-monitoring sites across four multiweek measurement periods. The study sites comprised two normally occupied residences and a test house. Measurement campaigns were conducted in summer and in winter. We report on the time-dependent concentration profiles of organosilicon compounds indoors and investigate factors influencing these concentrations, including the respective sources.

## Materials and Methods

### Measurement Methods

Volatile organic compounds (VOCs), including VMS and other organosilicon species reported in this paper, were measured by a proton-transfer reaction time-of-flight mass spectrometer (PTR-TOF-MS), manufactured by Ionicon, during observational and experimental campaigns described in the next section. Details of how this instrument works and how VOCs were identified and quantified are reported in Liu et al.^40^ Briefly, the sampled VOCs react with a hydronium ion (H_3_O^+^) to become protonated. The positive charge allows for the VOCs to be accelerated electrostatically in the mass spectrometer’s time-of-flight chamber. The time needed for a protonated molecule to traverse the time-of-flight chamber determines the mass-to-charge ratio (*m*/*z*) of the detected compound and the associated signal intensity determines that compound’s concentration. More details can be found in Holzinger.^41^ The *m*/*z* ratio specifies a chemical formula, which can sometimes lead to compound identification. More details about the instrument and detected organosilicon compounds can be found in the Supporting Information (Table S1).

### Observational Campaigns

#### House 1 (H1)

Two intensive indoor air monitoring campaigns were conducted at H1, a normally occupied two-level single-family dwelling in Oakland, California. Data collection occurred during 8 weeks starting in mid-August 2016 (H1 summer, “H1S”) and during 5 weeks starting in late January 2017 (H1 winter, “H1W”). Six monitoring locations were used for most of the campaigns: the attic, basement, crawlspace, the landing outside the bedrooms on the upper level, the kitchen on the lower level, and outdoors. For this site, the volume-weighted living zone concentration was assessed using data from the landing outside of the bedrooms, which was assumed to represent the upper level with an estimated volume of 150 m^3^, and the kitchen, which was assumed to represent the lower level with an estimated volume of 200 m^3^.^40^

A valve was set to change sampling location every 5 min for the PTR-TOF-MS, with a full six-location cycle conducted twice per hour. Each perfluoroalkyl (PFA) sampling tube was 30 m long.^40^ To ensure that the valve-switching process did not influence results, the first 2 min of each 5-min sampling period were discarded. The remaining 3 min of each sampling period were then averaged during analysis. For some species, the first 4 min were discarded due to the time required for a compound to equilibrate after valve-switching. To generate the average living zone concentration profiles, the data from each sampling location was linearly interpolated at 5 min resolution, then averaged. The interpolation ensured there would be a numerical value for each location at each time step to enable averaging.

Site H1 had two adult occupants with occasional guests during both measurement periods. Occupants were absent for a week at the end of the summer campaign and for a few days at the beginning of the winter campaign to enable comparison of indoor air composition when the house was occupied vs. unoccupied. In addition to VOC measurements, metadata sensors were placed throughout the house to monitor temperature, humidity, motion, and appliance usage, and the occupants maintained a detailed presence and activity log. Results of selected aspects of the H1 campaign have been previously reported, respectively, focusing on ventilation and interzonal airflows,^42^ VOC concentrations and dynamics,^40^ and ozone-initiated indoor chemistry.^43^

#### House 2 (H2)

The H2 (“H2W”) campaign entailed multiweek observational monitoring of a normally occupied single-family home during a winter period. Details are reported in Lunderberg et al.^39^ and Kristensen et al.^44^ This campaign was conducted in a single-story California ranch-style house in Contra Costa County, California. Data collection occurred over 9 weeks starting from December 2017 with monitoring sites in the attic, hallway adjacent to bedrooms, crawlspace, kitchen, living room, and outdoors. For this campaign, the living zone was determined from concentration measurements in the hallway, kitchen, and living room. The total estimated volume was 380 m^3 ^^44^ and each sampling location was weighted equally in the analysis of living zone concentrations discussed in this paper. As at H1, a valve was used to switch the PTR-TOF-MS among sampling locations, with the same tubing material and length as at H1,^39,40^ every 5 min, with a complete cycle requiring 30 min. The data for each living zone sampling location were linearly interpolated at 5 min resolution and averaged to obtain the living zone concentration time series. There were two adult occupants and one teenage occupant plus occasional guests. The occupants maintained a detailed presence and activity log during the monitoring campaign. The occupants were away from the house for 1 week during the monitoring campaign, allowing for an occupied vs. unoccupied comparison, and metadata sensors were placed throughout the house to monitor temperature, humidity, motion, and appliance usage.

### HOMEChem Experiments

The HOMEChem experiments were conducted in June 2018 in a test house on the J.J. Pickle Research Campus at the University of Texas, Austin. In contrast to the observational campaigns at H1 and H2, HOMEChem was designed with scripted indoor activities. Details have been reported by Farmer et al.^45^ and Arata et al.^46^ Experiments consisted of repeated indoor activities (e.g., cooking, cleaning), variable occupancy, and day-long sequences of “typical” indoor behavior. Two experimental days simulated the “Thanksgiving holiday,” which entailed a small group of volunteers cooking food typically served at Thanksgiving for several hours and a larger group entering later in the day to join in eating the meal. On two experimental days, sampling was conducted while the test house was vacant. VOCs were sampled with the PTR-TOF-MS from the kitchen as well as outdoors, with a valve set to switch from the indoor location to the outdoor location after 25 min and back to the indoor location after 5 min, for a complete cycle of 30 min. In this study, PFA sample tubing was 8.4 m.^46^ In data analysis, the first 5 min of each indoor sampling period and the first 4 min of each outdoor sampling period were discarded. Data were interpolated linearly at 1-min resolution. Sensors inside the test house measured metadata in a manner similar to the H1 and H2 studies.

## Results and Discussion

### Concentrations of Organosilicon Species

This section presents summary statistics on concentrations of organosilicon species measured during the four monitoring campaigns. We provide analysis of the normally occupied residence data and the HOMEChem data separately. Table S2 reports the living zone concentrations of organosilicon compounds measured during three monitoring campaigns in the ordinarily occupied households: H1 summer, H1 winter, and H2 winter. (Some data for H1 summer and H1 winter are also found in Liu et al.^40^) The table presents the means, medians, and 75^th^ and 90^th^ percentile values, determined from the average living zone concentrations at 5 min resolution, for each organosilicon compound measured, with separate entries for the occupied (O) and vacant (V) periods.

In each campaign, all four cyclic VMS species (D3-D6) are present, with D5 siloxane being the most abundant for both occupied and vacant periods. All four were present at median levels spanning less than an order of magnitude during the occupied period in the summer, with vacant period concentrations being substantially lower. During the winter periods at both H1 and H2, the median D5 siloxane concentrations were 7× and 19× higher than the summer campaign (H1S), respectively. Interestingly, the median D5 concentration at H2W during the vacant period was 4× that measured during the vacant period for H1W. Also worth noting is that the 90th percentile concentration during the occupied periods was about 3 times higher than the median during H1S and H2W, but a factor of 12 higher for H1W. The relatively large variance in indoor concentrations reflects the dominant effect of episodic release events that are mainly associated with personal care product use. Following D5, the next most abundant cVMS was D4 siloxane, which showed only small changes in median levels comparing occupied and vacant periods. D3 and D6 were present at median levels in the range 0.01–0.1 ppb during the occupied period and were moderately lower during the vacant period. Occupancy was generally associated with increased cyclic siloxane abundance indoors. Outdoor summary statistics showed that concentrations for all three measurement periods were at least an order of magnitude lower than indoors or below the reporting limit of 0.005 ppb.

Of the remaining five compounds considered, none were detected during the H1S monitoring period and only silyl acetate was detected in the H1W monitoring period. On the other hand, the full suite of nine organosilicon species was detected in the H2W monitoring campaign. Excluding cVMS, silyl acetate had the highest abundance, followed by L4, L5, and C7, which had concentrations around a factor of 2 lower. Concentrations during the vacant period were consistently lower than for the occupied period at H2W, as was also seen for silyl acetate in H1W. Caprylyl methicone concentrations were just above the reporting limit in both the occupied and vacant periods, with the occupied versus vacant comparison suggesting that occupancy does not strongly affect emissions of this compound.

**Figure 1:**
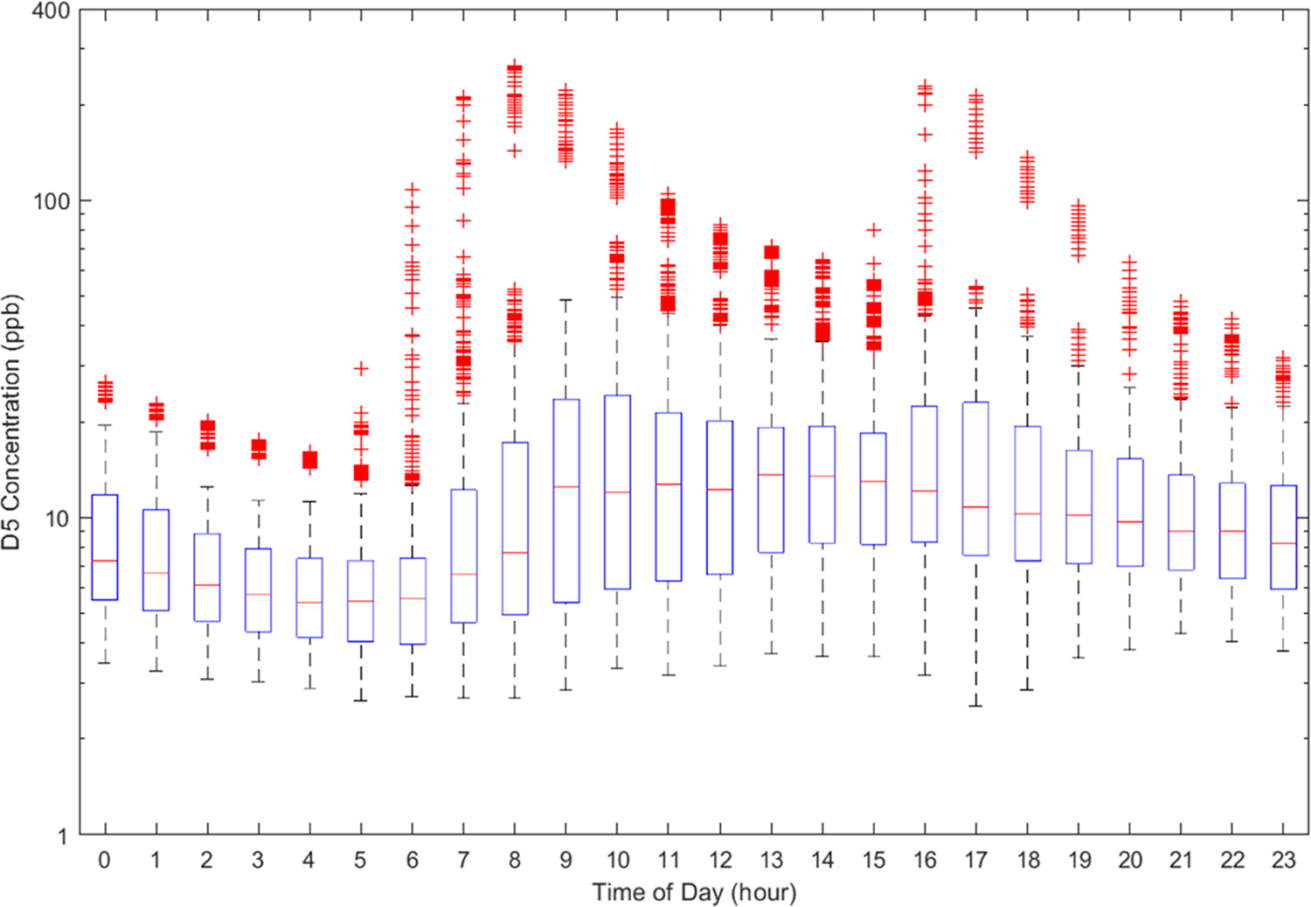
D5 siloxane diurnal plot in the living space during occupied period of the H2W campaign. Top and bottom edges of the blue boxes represent the 75th and 25th percentiles, respectively, and the red lines inside the boxes represent the medians. The black whiskers extend distances of 1.5 times the interquartile range from the box edges. The red symbols denote positive outliers.

Figure 1 displays a diurnal plot of D5 siloxane concentrations averaged throughout the living zone during the occupied period of the H2W monitoring campaign. The upper range of outliers relative to the central tendency clearly demonstrates the wide variability of daily D5 concentrations, which is a consequence of the timing and quantity of D5-containing personal care products applied. Specifically, a morning peak is evident at 7:00–8:00, when occupants would typically apply personal care products. A subsequent decay is observed throughout the day when the occupants were often away at work or school. A late afternoon peak appears in the outliers, around 16:00–17:00, when occupants returned home and may have heavily reapplied their personal care products on certain days. In central tendency, it appears likely that afternoon increases are linked to the return of an occupant and not necessarily associated with reapplication of products. Peak D5 concentrations are as high as 250 ppb, much higher than for any of the other organosilicon compounds measured.

Table S3 presents the mean concentrations of detected compounds for each experiment conducted at HOMEChem. (Some prior reporting of these data are in Farmer et al. and Arata et al.^45,46^ Averaging time periods were based on the length of each scripted experiment during the campaign.^45^) All four cyclic VMS and silyl acetate were above the reporting limit in every experiment. L4 was detected during a layered day experiment, an occupancy experiment, and the “open house”. Caprylyl methicone was detected during a “Thanksgiving” experiment, an unoccupied experiment, and a cleaning experiment, as well as during the open house and enhanced ventilation experiments. L5 was only detected during the open house at a concentration near the reporting limit, and C7 was not detected. The higher average signals during the open house, as well as the wider variability of compounds detected, are likely a result of higher occupancy and diversity of personal care products used. However, because the record of personal care product usage at HOMEChem is limited, this feature of the study cannot be further explored.

As in the monitoring campaigns at H1 and H2, D5 siloxane was the most abundant species for all HOMEChem experiments. Excluding the simulated Thanksgiving experiment, the other cyclic species exhibited mean concentrations in the respective ranges 0.03–0.08 ppb for D3, 0.03–0.10 ppb for D4, and 0.05–0.19 ppb for D6. The Thanksgiving experiments tended to have higher mean levels of D3, D4, and D6 siloxanes than the other HOMEChem experiments (see the SI for more information). Mean D5 levels tended to be highest in the experiments with high occupancy: Thanksgiving (4.5 and 4.1 ppb), occupancy (5.8 ppb), and open house (5.9 ppb). These findings are consistent with expectations that personal care products are the major indoor source of D5. Outdoor concentrations (not displayed) were consistently much lower than indoors and often below the reporting limit of 0.005 ppb.

Considering average concentrations across the different monitoring campaigns, the D5 concentration was highest in the two observational monitoring campaigns conducted during winter, with mean values of approximately 15 ppb. There is substantial variability in concentrations across individual days of the campaigns. The average D5 concentrations during H1W and H2W are about a factor of 3 higher than those measured during the Thanksgiving experiments at HOMEChem. This may be because occupants in H1S, H1W, and H2W applied their personal care products at home, whereas participants in HOMEChem typically applied their products elsewhere before arriving at the test house. The average D5 level at H1W is ∼16× higher than in H1S. Neither linear siloxane was present at the HOMEChem Thanksgiving. Average caprylyl methicone concentrations during HOMEChem Thanksgiving were consistent with those found at H2W, an unexpected finding given the greater occupancy level throughout this HOMEChem experiment, presumed higher aggregate personal care product usage during the Thanksgiving experiments, and wide array of products containing this compound.^47^ More information regarding cVMS during these Thanksgiving experiments can be found in the SI.

### Dynamic Behavior and Source Attribution for Cyclic Volatile Methyl Siloxanes in H2

In this section, we present a detailed analysis of cVMS emission events during the H2W campaign, emphasizing source attribution and average rates of emission and decay for the living zone concentration time series. The decay rates are compared to the average air-change rate of H2 to establish whether ventilation is the dominant removal mechanism in the living zone. We also provide information on co-occurrence of separate species during major peaks to determine the variability in sources of episodic emissions, as sources may contain different cVMS mixtures. Finally, the properties of episodic cVMS peaks are examined to provide insight on the variability of events, which could also be attributed to differing compositions of emission sources. The time series data presented in this and in the linear VMS section represent the average living zone concentration and outdoor concentration profiles from the H2W campaign. Details are presented in the SI.

**Figure 2:**
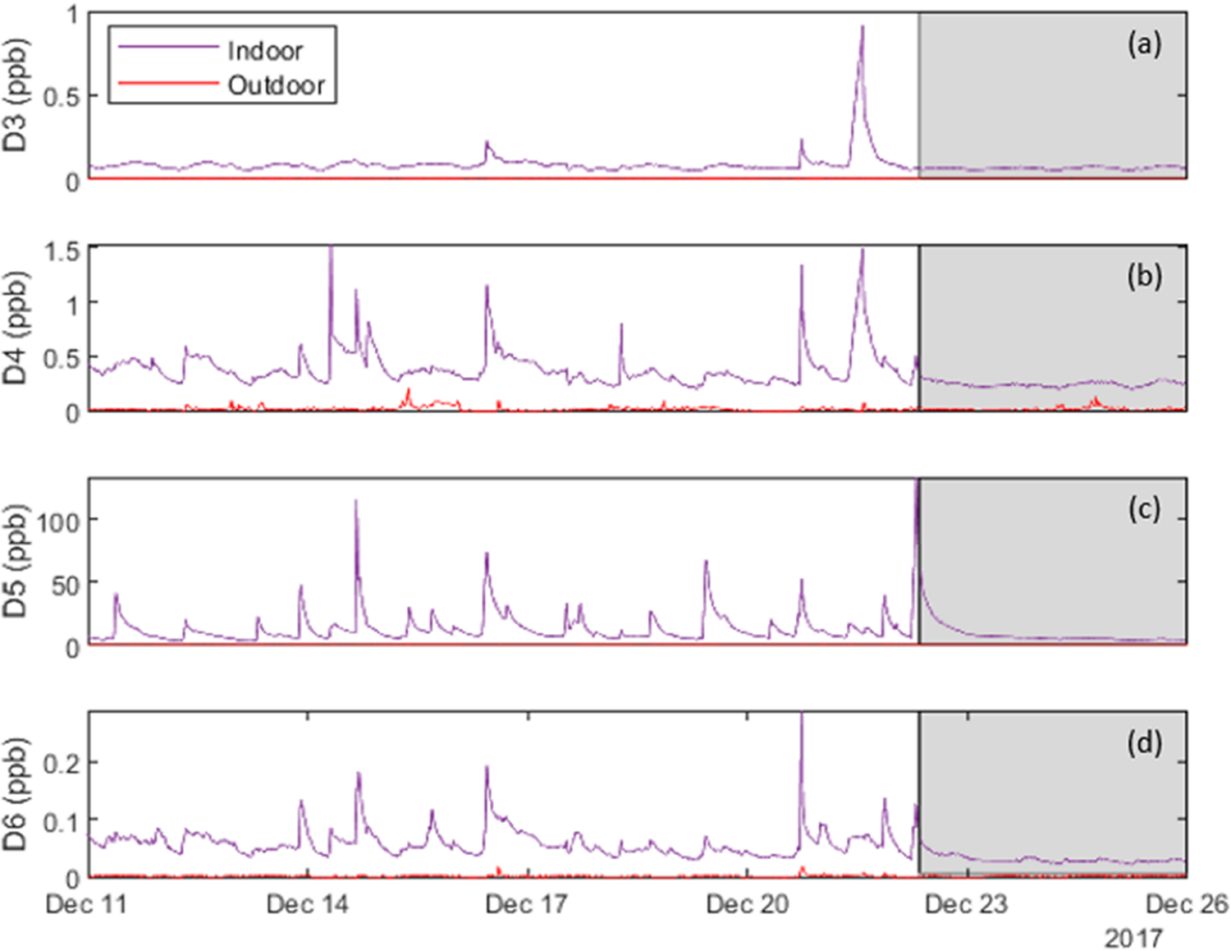
Indoor and outdoor cyclic VMS concentration time series measured during a portion of the H2W campaign for (a) D3 (previously reported in Lunderberg et al.^39^), (b) D4, (c) D5, and (d) D6. The shaded region, which represents the vacant period, exhibits no episodic concentration spikes.

Figure 2 shows time series measured during a portion of the H2W campaign of the indoor (purple) and outdoor (red) concentrations of four cyclic VMS: (a) D3, (b) D4, (c) D5, and (d) D6. The indoor concentration profile represents an average of measurements taken at the three living zone sampling sites: the living room, the kitchen, and the bedroom. Profiles are at 5 min resolution. Indoor concentrations are consistently higher than outdoor concentrations, indicating the dominant role of indoor emissions affecting indoor concentrations. All profiles show daily fluctuations in baseline concentrations. Peaks in each indoor trace are caused by episodic emission events, with varying levels of co-occurrence. Based on visual examination of the time-series plots for the full H2W campaign, we identified and analyzed seven D3 peaks, 21 D4 peaks, 44 D5 peaks, and 15 D6 peaks. Each of the D3 peaks coincided with a D4 peak, suggesting that D3 was always emitted from a source that also contained D4. The average ratio of mass of D3:D4 emitted during these emission events was 0.28 ± 0.15. Only two D3 emission events coincided with D5 and D6 emission events. Ten D4 emission events (half of the total) coincided with D5 events, and eight coincided with D6 emission events. Siloxane D5 showed a higher co-occurrence rate with D6 than with the other cyclic compounds, as 11 emission events (a quarter of all D5 events and three-quarters of the D6 events) occurred simultaneously. More specific information about event co-occurrence can be found in Table S6.

Siloxane D5 is the most abundant VMS species at H2. Next in abundance was D4, which is known to be in personal care products and adhesives.^19^ One especially prominent D4 emission event occurred on 26 January 2018, accounting for half of the total mass emitted during the H2 campaign. Other siloxanes show a spike at the same time (approximately 11:30), and motion sensors report movement around the time of the emission event, indicating indoor human activity; however, no specific product use was reported in the activity logs. On average, as noted in Table S2, the concentration of D4 was higher than those of D3 and D6 siloxanes, which were present at concentrations of the same order of magnitude. From this information alone, it is difficult to say whether these compounds are continuously emitted from the same source in the house. However, as shown in Figure 2, concentration spikes are not always coincident, indicating that they have separate episodic sources that are probably distinct from their continuous source(s). Siloxane D6 is known to be in cleaning products,^40^ and while D3 may also be present in those products, it must have at least one other significant source.

Table S4 summarizes cVMS source attribution, average emission and decay rates, and peak properties. The explanation of how this table was developed is provided in the SI. Contributions to indoor cVMS levels from the outdoors were minor, as expected. Indoor concentrations of cyclic siloxanes D3, D4, and D6 are dominated by continuous sources, a finding based on the low frequency of emission events and by the relatively small masses emitted during these events. Results for D5 siloxane indicate that both episodic and continuous sources contribute substantially to total indoor emissions.

D5 siloxane is emitted into H2 at a much higher rate than the other cVMS species. The decay rates of each compound are similar to the residence average air-change rate, which suggests that ventilation is the dominant removal mechanism for these compounds. However, the range of decay rates indicate the possibility that ventilation does not always dominate (see the Supporting Information). In some cases, decay rates are high enough to bring into question whether these compounds undergo substantial reversible sorption onto accessible surfaces, which could affect the categorization of emission sources. Statistical information on the distribution of decay rates for each compound is reported in Table S5. Information on H1S and H1W source attribution, emission rates, and peak properties are found in Table S7.

### Dynamic Behavior and Source Attribution for Linear and Other Organosilicon Species in H2

**Figure 3:**
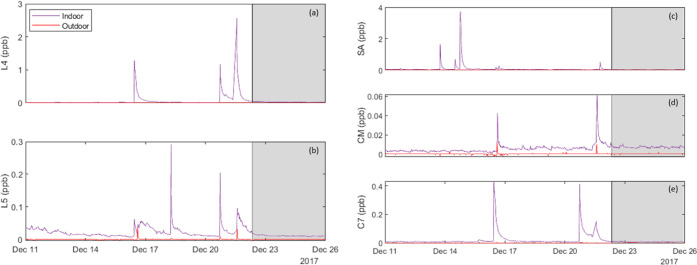
Indoor and outdoor concentration time series at H2 for five species: (a) L4, (b) L5, (c) silyl acetate (SA), (d) caprylyl methicone (CM), and (e) C_7_H_20_O_3_Si_3_ (C7). The shaded region represents the vacant period.

In this section, we summarize emissions assessments for the linear VMS and remaining organosilicon species. Figure 3 (left frames) shows time series from a portion of the H2W campaign of the living space and outdoor concentrations for two linear siloxane species, (a) L4 and (b) L5. Nine peaks in the L4 profile and 15 peaks in the L5 profile were identified. Six of these emission events appeared to occur simultaneously for L4 and L5. Much like the cyclic species, peaks in the two species do not always co-occur, indicating that some emissions are from distinct sources. An additional point of interest is the duration of the decay interval for some peaks: the persistence above background levels can span multiple days, much longer than would be the case for removal of a conserved (nonsorbing) species by ventilation alone. This point is explored further in Figure S2.

The right side of Figure 3 shows concentration time series for three other silicon-containing species, whose *m*/*z* ratios correspond to the formulas for (c) silyl acetate (SA, C_2_H_6_O_2_Si), for which 22 emission events were identified; (d) caprylyl methicone (CM, C_15_H_38_Si_2_O_3_), for which two events were observed; and (e) an unidentified seven-carbon species (C_7_H_20_O_3_Si_3_), for which seven events were detected. Silyl acetate is an ingredient in acetone-based nail polish remover and caprylyl methicone is used in a variety of personal care products, including some cosmetics and sunscreens.^39,47,48^ None of the emission events for these three species appeared to occur simultaneously, indicating distinct primary sources.

To our knowledge, measurements of silyl acetate in indoor air have not been previously reported. Both caprylyl methicone and the unidentified C7 compound were previously attributed to indoor sources at H2,^39^ but to our knowledge have not been reported in any other indoor air study. Silyl acetate was present in the H1W campaign, as indicated in Table S2. Silyl acetate and caprylyl methicone were both detected in several experiments at HOMEChem, but the unidentified C7 compound was only above the reporting limit of 5 ppt at the HOMEChem site during the Thanksgiving experiments, as indicated in Table S3. Not enough is known about the C7 compound to suggest a specific source.

Total indoor emissions of L4 and L5 over the H2 campaign were 106 mg and 95 mg, respectively. Transport from the outdoors accounted for less than 1% of these totals. Average indoor emission rates were 1.8 mg/d and 1.6 mg/d for L4 and L5, respectively. Throughout the campaign, it appeared that some episodic emission events in the linear VMS profiles took more than a day to return to steady state rather than several hours, as would be expected if these compounds were being removed primarily by ventilation. To illustrate, Figure S2 presents a log-linear time-series plot for L5 for a 2-day period beginning at midnight on 18 December 2017. The purple trace shows measured concentration data, whereas the orange line indicates the expected behavior from the measured peak in the case of first-order decay by means of ventilation at a rate of 0.5 h^–1^. After peaking, the measured concentration initially decays more rapidly than for removal by ventilation only, and subsequently decays much more slowly.

One interpretation of this evidence is that L5 partitions substantially, but reversibly, to indoor surfaces. Following an episodic indoor release, most L5 is temporarily lost to indoor surface reservoirs, rather than being removed by ventilation. Over time, as ventilation reduces the airborne concentration, surface-sorbed L5 is slowly reemitted, leading to the exhibited persistence of elevated concentrations. This interpretation could account for the consistently elevated levels over the course of several days following episodic emission events. The time pattern of concentrations would be pertinent for efforts to understand the influence of indoor emissions on exposures in the case of intermittent occupancy. It also complicates the efforts to perform a more detailed source apportionment as was done for cVMS species. However, such an apportionment was made for the remaining three organosilicon species, as reported in Table S4.

### Comparing Species across Campaigns

**Figure 4:**
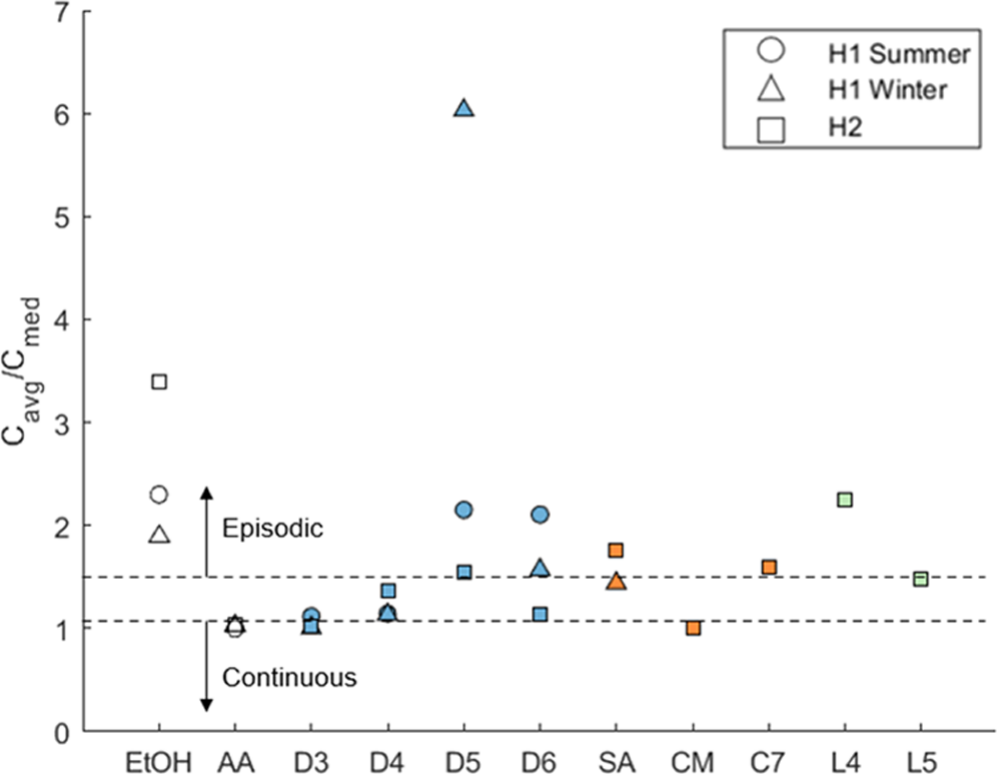
Mean-to-median ratios of ethanol (EtOH), acetic acid (AA), and the nine siloxane species in the H1S, H1W, and H2W campaigns. MMR for H1S and H1W can be found in Liu et al.^40^ Colors differentiate species, with white indicating nonorganosilicon species, blue indicating cVMS, orange indicating organosilicon species, and green indicating linear VMS.

This section compares each compound’s abundance and behavior across campaigns. It has been reported that some indoor air contaminants are dominated by episodic emissions, whereas others are dominated by continuous emissions.^40,49^ In this study, not all species are consistently dominated by the same category of emissions across campaigns, a finding illustrated in Figure 4. This figure shows a scatter plot of the concentration mean-to-median ratios (MMR), color-coded by species, and with marker shapes identifying the respective campaigns. Plotted are MMR values for 11 species: ethanol (which is consistently dominated by episodic emissions), acetic acid (which is consistently dominated by continuous indoor emissions), and the nine organosilicon species discussed in this paper. An important distinction to note is that the MMR is calculated using concentrations from the kitchen sampling locations during the occupied periods of H1S, H1W, and H2W only, to be consistent with Liu et al.^40^ The lower dashed line is drawn at a ratio of 1.06, which is the upper limit proposed by Liu et al.^40^ for species dominated by continuous emissions. The upper dashed line is drawn at a ratio of 1.5, which is the lower bound for episodic emissions dominance. An MMR ratio between 1.06 and 1.5 indicates that both continuous and event-driven emissions contributed materially to indoor concentrations. As indicated in this figure, D3 and caprylyl methicone would be categorized as continuously emitted compounds in all three campaigns; D4 is consistently in the band between the continuous and episodic thresholds; D5, C7 (C_7_H_20_O_3_Si_3_), L4, and L5 are episodically emitted compounds; and D6 and silyl acetate vary in their categorizations among the three campaigns.

Across all campaigns, the most abundant organosilicon species was D5. Emissions of this compound were primarily episodic, with emissions occurring daily and with relatively consistent decay behavior following episodic peaks. Interestingly, the two H1 campaigns had starkly different average D5 concentrations, suggesting wide variability in the amount or type of product used across seasons despite having the same household occupants. Although the average air-change rate during H1S was higher than H1W,^42^ both D3 and D6 siloxane concentrations were higher during H1S. The high average D5 concentration at the H2W campaign was expected due to some of the occupants’ heavier personal care product usage. The Thanksgiving experiments in HOMEChem might be expected to have high D5 concentrations because of higher occupancy in the test house than on most of the other experimental days, but they did not approach the levels found during H1W and H2W. Evidently, the specific personal care products used as well as the total amounts applied are more important than the number of occupants. Additionally, participants likely applied their personal care products before arriving at the test house. The other three cyclic VMS species investigated did not exhibit consistent categorization, indicating heterogeneity in emissions sources.

Observations of emission event coincidence during H2W for the different organosilicon species provides some insight into variability in composition of observed sources. Every D3 siloxane peak, along with seven D4 peaks, coincided with L4 and C7 peaks, indicating likely commonality in episodic emission sources. One of the coincident D5 and D6 peaks also occurred simultaneously with L4 and C7 events, implying either a second source of these compounds or the presence of a source of D5 and D6 only that happened to emit simultaneously. Similar implications emerge when comparing the coincidence of different species with L5 siloxane. Interestingly, only one silyl acetate peak coincided with any other compound (D5 siloxane), indicating that silyl acetate has at least one source that is independent of all of the other organosilicon compounds examined. The variability in potential episodic emission sources implied by this brief analysis does not appear to be present at H1, given that only a subset of the organosilicon compounds explored in this paper were observed. More information regarding the number of compounds emitted during simultaneous emission events at H2 is found in Table S6.

D3 was characterized as having a mostly constant background emission source with occasional event-driven emissions. Some emission events occurred at the same time for more than one siloxane species, implying that some products contain multiple siloxanes, whereas other events occurred independently for the different siloxanes (see the SI for more information). The additional indication of potential temperature-driven behavior in the case of D3 and D4 prompts the discussion of what other materials and products contain siloxanes and what drives their emissions. D4 siloxane shows evidence of both event-driven emissions and background sources across campaigns. This could potentially be explained by two known source categories: adhesives,^19^ which contribute to VOC emissions upon application and show evidence of diffusion-controlled emissions after application,^50^ and personal care products, which should mainly manifest in episodic emissions. However, there is some uncertainty due to the variability in housing materials and occupant products. D6 emissions appear to be primarily event-driven in both seasons of H1, but primarily background in H2, which can also be a result of material and product variability. Additionally, the average concentration of D6 was higher on both Thanksgiving days in HOMEChem than in H1S, H1W, or H2W, further indicating that even on individual days, there is substantial variability in usage of products containing D6 siloxane (and others) and, consequently, in abundance of D6 in the air.

Emissions of both L4 and L5 were primarily event-driven in H2. These species showed much lower average abundance during the HOMEChem Thanksgiving days than at H2, once again pointing to the importance of the types of products in use. Neither compound exhibited average concentrations above the 5 ppt reporting limit during either of the H1 campaigns. Although not as ubiquitous or abundant as the cyclic VMS compounds, L5 siloxane appears to persist indoors longer than its cyclic counterparts, which may have implications regarding surface interactions and for human exposure. Silyl acetate is primarily event-driven in H2, shows some background sources in H1W, and was measured above the reporting limit in HOMEChem. One identified source of this compound was acetone-based nail polish remover, used in H2. Caprylyl methicone emissions are primarily from background sources in H2 but appear to be event-driven in HOMEChem, possibly corresponding to sunscreen or cosmetic use during that campaign. The remaining organosilicon compound, C_7_H_20_O_3_Si_3_, is event-driven in H2, but no product has been confirmed to contain it, and it did not exceed the reporting limit in either season of H1 or in HOMEChem.

This analysis has combined measurement results with analysis for nine organosilicon compounds from four multiweek indoor air monitoring campaigns. Three observational campaigns were conducted in normally occupied residences; the fourth was undertaken at a test house with scripted experiments. Siloxane D5 was the most abundant organosilicon species across campaigns. Siloxanes D3 and D4 were found to be emitted by sources other than personal care products. Linear siloxanes L4 and especially L5 showed evidence of reversible sorption that strongly affected the time pattern of concentrations following episodic release events, despite having vapor pressures similar to D5 and D6 siloxanes, respectively. This study has also reported measurements of three organosilicon compounds that have not been extensively discussed in the literature; two of them had identifiable sources, while the origin of the third remains unidentified.

## Supporting Information Available

PTR-TOF-MS operation and QA/QC; organosilicon compound identification and properties; source apportionment, emission/decay rate, and emission statistics; time series; mass balance; and emission event co-occurrence (PDF)
