# Supplementary material for: Volatile Methyl Siloxanes and Other Organosilicon Compounds in Residential Air
Source: Environ Sci Technol. 2022 Nov 3;56(22):15427–36. doi: 10.1021/acs.est.2c05438 (PMC9670844; doi:10.1021/acs.est.2c05438)
Supplement: Supplementary file 1 — es2c05438_si_001.pdf [file es2c05438_si_001.pdf]

Supporting Information for

Volatile Methyl Siloxanes and Other Organosilicon Compounds in Residential Air

Betty Molinier[1], Caleb Arata[2,3], Erin F. Katz[2,3], David M. Lunderberg[2,3], Yingjun Liu[3,4], Pawel K. Misztal[3,5], William W Nazaroff[1], Allen H. Goldstein[1,3]

[1]Department of Civil and Environmental Engineering, [2]Department of Chemistry, and [3]Department of Environmental Science, Policy and Management, University of California, Berkeley, California 94720, United States, [4]College of Environmental Sciences and Engineering, Peking University, Beijing 100871, China, [5]Civil, Architectural, and Environmental Engineering, University of Texas at Austin, Austin, Texas 78712

SI Contains:

17 pages

8 tables

5 figures

SI Table of Contents

1. (*page S3*) - Measurement Methods
   a. **Table S1.** Physicochemical properties of detected organosilicon compounds at 298 K.
   b. **Figure S1.** Chemical structures.
2. (*page S5*) – Concentrations of Organosilicon Species
   a. **Table S2.** Mean, median, 75th percentile, and 90th percentile concentrations (ppb) of each reported organosilicon compound measured in the living zone during three observational campaigns, separately considering occupied (O) and vacant (V) periods.
   b. **Table S3.** Mean concentrations (ppb) of each reported organosilicon compound indoors during various HOMEChem experiments (number of replicates).
3. (*page S7*) - Dynamic Behavior and Source Attribution for Organosilicon Species in H2
   a. **Table S4.** Source attribution (%), average emission and decay rates, and episodic mass emission statistics (mg) of detected cyclic siloxanes and organosilicon compounds in the living zone of H2 during the occupied period.
   b. **Table S5.** Decay rate coefficients (h^{-1}) for organosilicon compounds at H2.
   c. **Figure S2.** Measured L5 concentrations before and after an emission event at H2.
4. (*page S10*) - Emission Event Co-occurrence at H2
   a. **Table S6.** Organosilicon species detected indoors at various co-occurring emission events throughout the H2 monitoring campaign.
5. (*page S12*) - Source Attribution and Peak Properties at H1
   a. **Table S7.** Source attribution (%), average emission rates, and episodic mass emission statistics (mg) of detected cyclic siloxanes and organosilicon compounds in the living zone of H1S and H1W during the occupied periods.
6. (*page S13*) - Source Attribution of D3 and D4 in 'Thanksgiving' Experiments at HOMEChem
   a. **Figure S3.** Time series of cyclic VMS during two HOMEChem 'Thanksgiving' experiments.
   b. **Figure S4.** Time series of D3 and D4 concentrations along with room temperature during HOMEChem Thanksgiving experiments 1 (a) and 2 (b).
   c. **Figure S5.** Amount of D3 and D4 emitted during each Thanksgiving event in HOMEChem, apportioned by source category.
   d. **Table S8.** Source apportionment of D3 and D4 emissions during HOMEChem 'Thanksgiving' experiments.
7. (*page S16*) - References

Measurement Methods

Table S1 shows the chemical formulae, molecular weights, mass-to-charge (*m/z*) ratios of the parent ions, and some physicochemical properties of the organosilicon species measured to gain insight into how these compounds behave under typical residential conditions. Figure S1 shows the corresponding chemical structures. The *m/z* ratio of a detected compound is determined from the rate constant of the reaction with the hydronium ion (H_3O^+) and the time it takes the protonated molecule to traverse the time-of-flight chamber in the mass spectrometer. This value can vary by the order of 0.001 depending on how the proton-transfer-reaction time-of-flight mass spectrometer (PTR-TOF-MS) is calibrated.

The PTR-TOF-MS was calibrated daily with one of two mixtures of calibration gases [1]. For compounds included in these standards, such as D5 siloxane, sensitivities determined directly from the calibrations were applied to convert signal strength to concentration. For other reported compounds, such as L5 siloxane, a default sensitivity was applied, based on a nominal rate coefficient for reactions with H_3O^+. More details about calibration can be found in Liu et al. [1].

Table S1. Physicochemical properties of detected organosilicon compounds at 298 K [2-7].[*]

Name (Chemical Formula)	MW (g mol^{-1})	Measured *m/z*	Vapor Pressure, kPa	log(K_{oa})	log(K_{aw})
D3 ($C_6H_{18}O_3Si_3$)[a]	222.46	223.066	0.471	4.05	0.42
D4 ($C_8H_{24}O_4Si_4$)	296.62	297.082	0.121[†]	4.28	2.74
D5 ($C_{10}H_{30}O_5Si_5$)	370.77	371.092	0.021[†]	4.94	3.13
D6 ($C_{12}H_{36}O_6Si_6$)	444.92	445.088	0.003[†]	5.86[‡]	3.01[‡]
L4 ($C_{10}H_{30}O_3Si_4$)	310.69	311.132	0.05	4.66	3.45
L5 ($C_{12}H_{36}O_4Si_5$)[a]	384.84	385.14	0.006	5.29	4.12
CM ($C_{15}H_{38}O_2Si_3$)[b]	334.72	335.221	0.0006	-	-
SA ($C_2H_6O_2Si$)[b]	90.15	91.0212	17.9	-	-
C7 ($C_7H_{20}O_3Si_3$)[c]	236.49	237.118	-	-	-

[*]MW = molecular mass; *m/z* = mass-to-charge ratio of the protonated compound; K_{oa} = octanol-air partition coefficient; K_{aw} = air-water partition coefficient; CM = caprylyl methicone; SA = silyl acetate; C7 = 7-carbon silicon organic compound.

[†] Assessed at 296 K; [‡] assessed at 297 K.

[a] The log(K_{oa}) and log(K_{aw}) values were calculated using equilibrium partitioning information consistent with Weschler and Nazaroff [8].

[b] Not enough information was found to calculate log(K_{oa}) and log(K_{aw}).

[c] No literature information was found for this compound.

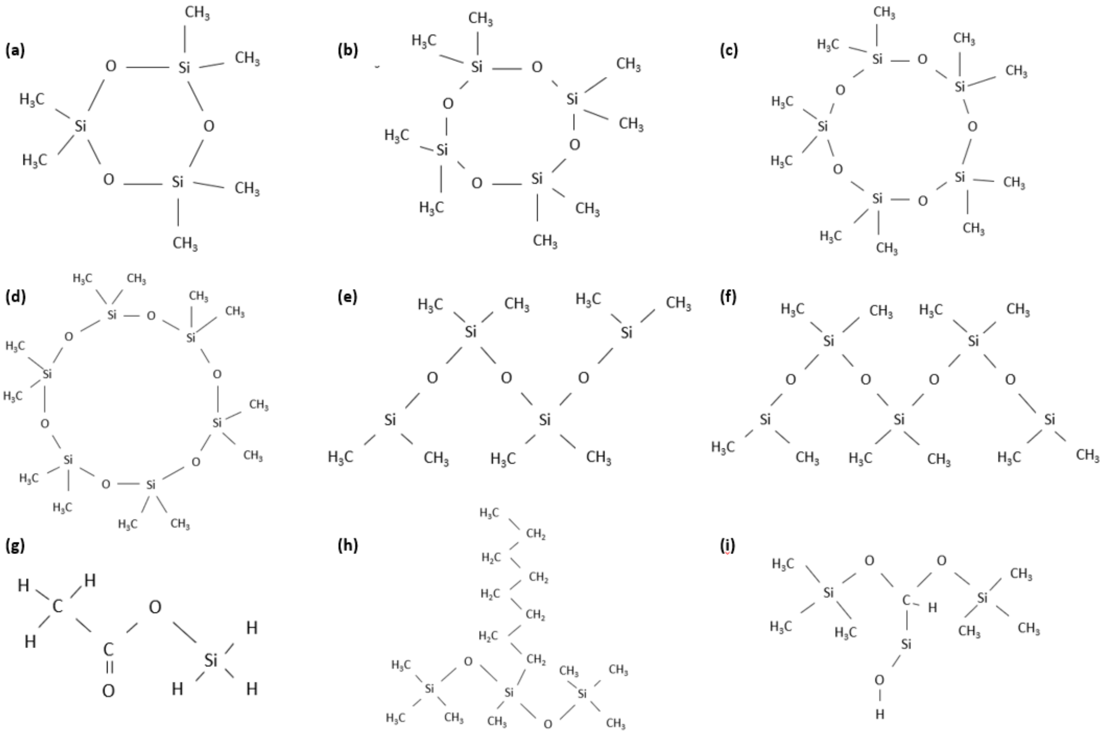

Figure S1. Chemical structures of (a) D3 siloxane, (b) D4, (c) D5, (d) D6, (e) L4 siloxane, (f) L5, (g) silyl acetate (SA), (h) caprylyl methicone (CM), and (i) $C_7H_{20}O_3Si_3$ (C7).

Concentrations of Organosilicon Species

This section presents summary statistics on concentrations of organosilicon species detected at each of the four monitoring campaigns. Table S2 provides mean, median, 75th, and 90th percentile concentrations in the living zone from H1 summer (H1S), H1 winter (H1W), and H2 winter (H2W). The vacant period refers to the multiday period in each campaign when the occupants were deliberately away, whereas the occupied period refers to the remaining time in each campaign. Days during which occupants were at work or otherwise outside of the house for several hours were included as "occupied" periods because of the influence of the occupants' activities in the morning before leaving or in the evening when returning to the house. Table S3 reports mean concentrations for each HOMEChem experiment.

Table S2. Mean, median, 75th percentile, and 90th percentile concentrations (ppb) of each reported organosilicon compound measured in the living zone during three observational campaigns, separately considering occupied (O) and vacant (V) periods.[a]

Field Site	Stats	**D3** $C_6H_{18}O_3Si_3$	**D4** $C_8H_{24}O_4Si_4$	**D5** $C_{10}H_{30}O_5Si_5$	**D6** $C_{12}H_{36}O_6Si_6$	**L4** $C_{10}H_{30}O_3Si_4$	**L5** $C_{12}H_{36}O_4Si_5$	**SA** $C_2H_6O_2Si$	**CM** $C_{15}H_{38}O_2Si_3$	**C7** $C_7H_{20}O_3Si_3$
**H1S**	Mean	O: 0.113<br>V: 0.082	0.112<br>0.077	0.89<br>0.43	0.130<br>0.037	-<br>-	-<br>-	-<br>-	-<br>-	-<br>-
	Median	O: 0.096<br>V: 0.084	0.093<br>0.078	0.44<br>0.41	0.058<br>0.028	-<br>-	-<br>-	-<br>-	-<br>-	-<br>-
	75th	O: 0.129<br>V: 0.095	0.122<br>0.086	0.70<br>0.44	0.190<br>0.030	-<br>-	-<br>-	-<br>-	-<br>-	-<br>-
	90th	O: 0.21<br>V: 0.107	0.180<br>0.096	1.35<br>0.47	0.33<br>0.038	-<br>-	-<br>-	-<br>-	-<br>-	-<br>-
**H1W**	Mean	O: 0.014<br>V: 0.007	0.23<br>0.28	14.5<br>1.03	0.059<br>0.011	-<br>-	-<br>-	0.076<br>0.035	-<br>-	-<br>-
	Median	O: 0.014<br>V: 0.007	0.20<br>0.26	3.0<br>1.05	0.038<br>0.011	-<br>-	-<br>-	0.054<br>0.034	-<br>-	-<br>-
	75th	O: 0.016<br>V: 0.008	0.26<br>0.28	7.8<br>1.11	0.058<br>0.012	-<br>-	-<br>-	0.068<br>0.038	-<br>-	-<br>-
	90th	O: 0.017<br>V: 0.008	0.34<br>0.37	37<br>1.18	0.096<br>0.013	-<br>-	-<br>-	0.118<br>0.041	-<br>-	-<br>-
**H2W**	Mean	O: 0.090<br>V: 0.060	0.48<br>0.23	13.4<br>6.3	0.055<br>0.027	0.037<br>0.019	0.027<br>0.009	0.084<br>0.031	0.006<br>0.007	0.023<br>0.008
	Median	O: 0.083<br>V: 0.058	0.36<br>0.23	8.3<br>4.3	0.047<br>0.026	0.013<br>0.016	0.018<br>0.009	0.045<br>0.031	0.005<br>0.007	0.010<br>0.008
	75th	O: 0.098<br>V: 0.065	0.45<br>0.25	14.7<br>5.3	0.064<br>0.028	0.017<br>0.022	0.032<br>0.011	0.058<br>0.034	0.007<br>0.007	0.012<br>0.008
	90th	O: 0.112<br>V: 0.071	0.61<br>0.27	25<br>8.3	0.084<br>0.033	0.048<br>0.027	0.050<br>0.012	0.102<br>0.036	0.009<br>0.008	0.024<br>0.009

[a] A dash indicates that the mean concentration of the compound was below the reporting limit of 0.005 ppb on a time-averaged basis.

To provide health-oriented perspective, the European guideline value of 0.4 mg m^{-3} for the sum of cVMS concentrations indoors [9] was applied as a benchmark, given that there are no such guidelines in the US. Using median concentrations, it was found that the sum of cVMS was less than this guideline at H1S, H1W, and H2W (0.01 mg m^{-3}, 0.049 mg m^{-3}, and 0.132 mg m^{-3}, respectively). Adding linear VMS and other organosilicon species did not alter this finding (no change in H1S or H1W; 0.133 mg m^{-3} at H2W). When using 90th percentile values, the outcome of the comparison changes: whereas the cVMS sum at H1S and H2W remained below the guideline (0.031 mg m^{-3} and 0.39 mg m^{-3}, respectively), even with the inclusion of additional

species, the summation at H1W was above the guideline value (0.57 mg m^{-3}). Consequently, under most conditions of this study, it appears that the use of personal care products containing siloxanes did not pose a significant health risk in the studied environments, according to this guideline.

Table S3. Mean concentrations (ppb) of each reported organosilicon compound indoors during various HOMEChem experiments (number of replicates)[a].

Experiment	D3	D4	D5	D6	L4	SA	CM
**Thanksgiving (2)**	0.133	0.110	4.5	0.62	-	0.77	0.005
	0.088	0.052	4.1	0.188	-	0.49	-
**Layered (4)**	0.041	0.028	0.88	0.082	-	0.043	-
	0.086	0.057	2.4	0.115	0.016	0.27	-
	0.068	0.047	1.95	0.099	-	0.44	-
	0.051	0.035	1.35	0.074	-	0.197	-
**Unoccupied (2)**	0.033	0.024	1.53	0.114	-	0.051	0.039
	0.066	0.037	0.85	0.053	-	0.030	-
**Stir Fry (2)[b]**	0.030	0.026	0.68	0.062	-	0.029	-
	0.044	0.033	1.07	0.053	-	0.032	-
**Cleaning (4)[c]**	0.027	0.022	0.74	0.050	-	0.028	-
	0.032	0.023	0.66	0.054	-	0.027	0.006
	0.042	0.030	1.04	0.061	-	0.039	-
	0.033	0.026	0.88	0.048	-	0.030	-
**Occupancy (3)**	0.029	0.025	0.54	0.056	-	0.026	-
	0.042	0.039	2.4	0.128	-	0.053	0.006
	0.114	0.23	14.4	0.24	0.042	0.055	0.047
**Open House (1)[d]**	0.079	0.069	5.9	0.189	0.012	0.073	0.037
**Enhanced Ventilation (1)**	0.032	0.027	0.48	0.070	-	0.050	0.013

[a] The average C7 concentration was below the reporting limit for all experiments.

[b] Measurement data were acquired for two of the three stir-fry experiments.

[c] Measurement data were acquired for four of the six cleaning experiments.

[d] L5 was present at 0.007 ppb during the open house but was otherwise below the reporting limit.

Dynamic Behavior and Source Attribution for Organosilicon Species in H2

In this section we present source attribution and decay rates of each examined organosilicon compound at H2. Measured time-resolved concentration profiles were used to assess emissions of their corresponding compounds over the duration of the H2W campaign. Total species mass emitted per interval (E_{tot}, g) was estimated from measured concentrations (C, ppb) at two-hour resolution using eq S1:

$$E_{tot} = MW\frac{P}{RT}V\left((C_{in}(t+\Delta t) - C_{in}(t)) + A\cdot\left(\overline{C}_{in} - \overline{C}_{out}\right)\cdot\Delta t\right)\times 10^{-9} \qquad \text{(S1)}$$

Here, MW is the compound's molecular weight, (P/RT) reflects the total molar concentration of air molecules (where P = 1 atm, T = 298 K), V is the volume of the house, and A is the average air change rate of 0.5 h^{-1} [10]. C_{in} and C_{out} refer to indoor and outdoor concentrations, respectively, with the overbars denoting averages over an analysis interval, from time t to $t + \Delta t$. The factor of 10^{-9} accounts for the concentration measurements in ppb. Air change rates were determined by releasing an inert tracer at a constant, known rate in the house, and measuring the time-dependent concentrations that result; those values are used in eq S2 to determine the time-varying air change rate [1].

$$A(t) = \frac{ER_{tracer}\Delta t - (C_{in}(t+\Delta t) - C_{in}(t))V}{\overline{C_{in}(t)}V\Delta t} \qquad \text{(S2)}$$

The parameter ER_{tracer} refers to the emission rate of the tracer continuously released into the living zone.

Equation S1 is useful for determining total emissions. A similar equation was applied for source apportionment. To that end, episodic peak emissions (E_{epi}) were estimated from eq S3 [11], which was obtained by subtracting emissions attributable to background sources from eq S1:

$$E_{epi} = MW\frac{P}{RT}V\left(\left(C_{in}(t+\Delta t) - C_{bkg}\right) + A\cdot\left(\overline{C}_{in} - C_{bkg}\right)\cdot\Delta t\right)\times 10^{-9} \qquad \text{(S3)}$$

Background sources are those that emit directly into the house, on a continuous basis, and not directly a result of occupant activities. Background concentrations are estimated as the average vacant period concentrations for each compound. Equation S3 was applied to the entire H2 concentration profile at a time resolution of two hours, and the mass emitted throughout the duration of each peak was recorded to evaluate the statistics presented in Table S4. Emissions during the episodic peaks were summed to assess the total percent contribution of episodic emission events. The contribution of continuous emissions was determined by subtracting outdoor contributions from the background, as in Lunderberg et al. [12]. Average emission rates were calculated by dividing the total amount of each compound emitted by the number of days of the campaign. Decay rates were determined by taking the natural log of the average indoor concentration profiles of each compound and using MATLAB's 'polyfit' function on each decay curve to extract the slope.

The emission characteristics of the cyclic VMS species were discussed in the main paper. Here, we discuss the properties of the three organosilicon species in more detail. As with the cVMS species, the outdoor contributions are negligible. Caprylyl methicone is dominated by emissions from a continuous source, while silyl acetate and C7 exhibit contributions of similar scale from episodic and continuous sources. Emission rates are lower for these compounds than for the cVMS. Caprylyl methicone and the C7 compound have emission rates in the range 0.1-1 mg/d, lower than

the other species studied. The observed decay rate of caprylyl methicone is higher than that of the other compounds, but as there were only two measured peaks, this finding is only suggestive that the high rate of decay is characteristic of this compound's indoor behavior. The average decay rate of C7 is lower than the average air change rate, whereas the average decay rate of SA is higher.

Table S4. Source attribution (%), average emission and decay rates, and episodic mass emission statistics (mg) of detected cyclic siloxanes and organosilicon compounds in the living zone of H2 during the occupied period[*,a].

**Compound**	**Average Emission Rate (mg d^{-1})**	**Source Attribution**			**Episodic Emissions**				
		Episodic	Continuous	Outdoor	Mean (mg)	Median (mg)	90th (mg)	DR[b] (h^{-1})	No. Peaks
**D3**	3.3	8%	90%	2%	2.2	1.9	3.8	0.41	7
**D4**	22	23%	73%	4%	14.5	6.3	16.9	0.66	21
**D5**	790	49%	51%	1%	520	320	1020	0.50	44
**D6**	3.9	16%	80%	3%	2.5	2.1	4.5	0.43	15
**SA**	1.20	45%	52%	3%	1.5	0.7	4.6	0.87	22
**CM**	0.34	2%	97%	1%	0.2[c]	-	-	1.73	2
**C7**	0.85	49%	49%	2%	3.5	2.4	7.9	0.37[d]	7

[*]Mean, median, and 90th percentile of the mass emitted during episodic events; DR = average concentration decay rate following episodic emission events; No. Peaks = number of peaks analyzed.

[a] The occupied period refers to these dates spanning 52 days: December 5-21 and December 29 - February 1.

[b] More information about decay rate distribution can be found in the Table S5.

[c] The two values were 0.09 mg and 0.3 mg.

[d] Only six of the seven analyzed peaks were used in this calculation.

The mean, median, 75th and 90th percentile, and the minimum and maximum observed decay rates for four cyclic volatile methyl siloxanes (cVMS) and all three additional organosilicon species are provided in Table S5. The linear VMS decay rates were not computed owing to strong evidence of reversible sorption, for which the decay behavior does not follow a first-order rate. D5 has a minimum decay rate (0.08 h^{-1}) that is much lower than the average air change rate (0.5 h^{-1}), and every species, with the exception of C7, has a maximum decay rate that is substantially higher. The former indicates the possibility of surface uptake, which Wang et al. [13] indicated occurs for D5, and the latter may be indicative of higher ventilation than the central tendency at H2 (for example, more windows than usual being opened).

Table S5. Decay rate coefficients (h^{-1}) for organosilicon compounds at H2.

Species	Mean	Median	75th	90th	Minimum	Maximum
D3 ($C_6H_{18}O_3Si_3$)	0.41	0.28	0.42	0.75	0.19	1.15
D4 ($C_8H_{24}O_4Si_4$)	0.66	0.38	0.65	1.52	0.11	2.4
D5 ($C_{10}H_{30}O_5Si_5$)	0.50	0.34	0.53	0.74	0.08	2.9
D6 ($C_{12}H_{36}O_6Si_6$)	0.43	0.35	0.46	0.72	0.23	1.10
SA ($C_2H_6O_2Si$)	0.87	0.76	0.86	1.46	0.35	2.6
CM ($C_{15}H_{38}O_2Si_3$)	1.73	1.73	2.3	2.6	0.70	2.6
C7[a] ($C_7H_{20}O_3Si_3$)	0.37	0.27	0.34	0.60	0.23	0.83

[a] Seven peaks were identified, but only six were used in calculating these statistics.

Figure S2 compares the actual indoor concentration profile (purple) for L5 over a 48-hour period to the modeled decay (orange) if the only removal mechanism was ventilation to the outdoors at an air change rate of 0.5 h^{-1}. Comparing the measured and modeled decay indicates that L5 initially is removed from indoor air more rapidly than by ventilation alone, and then subsequently decays more slowly. This behavior is consistent with the effect of ventilation being modulated by reversible sorption, e.g. by transient uptake in surface reservoirs.

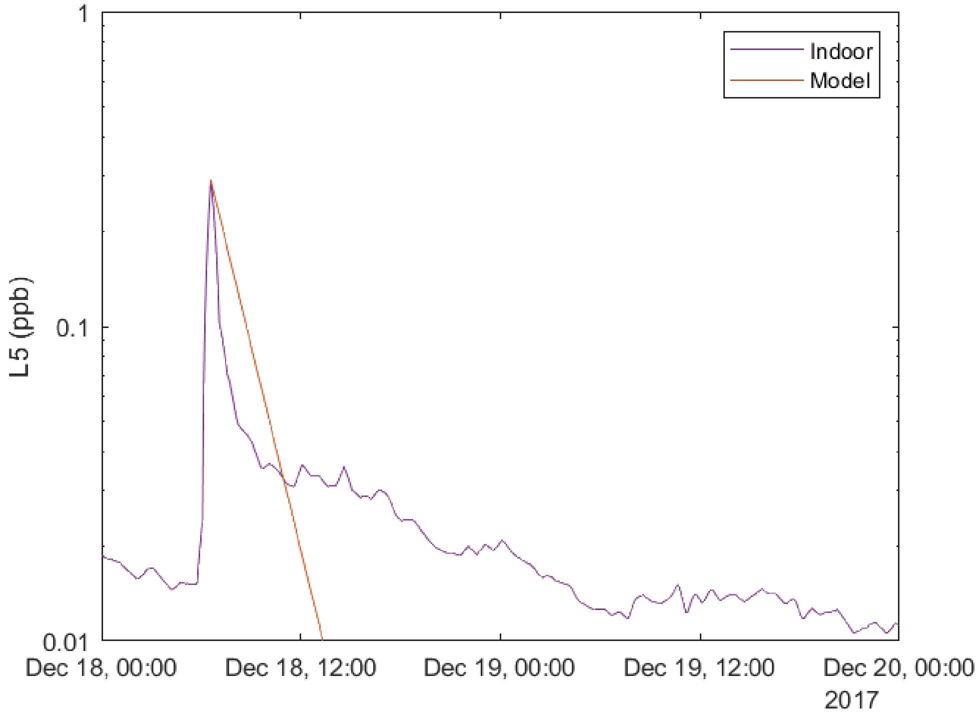

Figure S2. Measured L5 concentrations before and after an emission event at H2. The modeled trace shows the straight-line decay from the peak concentration that would occur if air-change at a rate of 0.5 h^{-1} were the only removal mechanism.

Emission Event Co-occurrence at H2

Instances of peak co-occurrence between organosilicon species, as well as unique single organosilicon species emission events, provide clues about source composition. Table S6 presents information on the combinations of species observed during emission events at H2. Personal care product usage was not recorded, and, consequently, many emission events could not be attributed to recorded activities. In addition to the four cyclic and two linear siloxane species, information on silyl acetate (SA), caprylyl methicone (CM) and an unidentified organosilicon species (C7) is presented. In two cases, the activity log indicates that no occupants were present, but motion sensor data indicates occupancy for both cases.

Table S6. Organosilicon species detected indoors at various co-occurring emission events throughout the H2 monitoring campaign.

Date and Time	No. Occupants Present	Activity	Compounds Detected
Dec 09, 2017, 17:35	2	Cooking	D5, L5
Dec 13, 2017, 21:05	3	Unknown	D4, D5, D6
Dec 14, 2017, 15:35	1	Unknown	D4, D5, D6
Dec 16, 2017, 9:05	4	Cooking	D5, D6
Dec 16, 2017, 10:05	5	Unknown	D3, D4, L4, L5, C7
Dec 16, 2017, 14:05	3	Unknown	L5, CM
Dec 18, 2017, 6:05	2	Unknown	D4, D5, L5
Dec 20, 2017, 17:05	2	Unknown	D3, D4, D6, L4, L5, C7
Dec 21, 2017, 9:05	2	Unknown	D3, D4, L4, C7
Dec 21, 2017, 13:35	6	Unknown	L5, CM
Dec 21, 2017, 20:05	3	Unknown	D5, D6
Dec 22, 2017, 5:35	0	Asleep	D4, D5, D6
Jan 02, 2018, 11:20	3	Unknown	D3, D4, L4, L5, C7
Jan 03, 2018, 7:40	1	Unknown	D4, D5, D6
Jan 05, 2018, 8:50	2	Unknown	D5, D6
Jan 09, 2018, 7:25	2	Unknown	D3, D4, D5, L4, L5, C7
Jan 10, 2018, 6:10	1	Unknown	D3, D4, D5, L4, L5, C7
Jan 13, 2018, 16:05	2	Unknown	D4, D5, D6
Jan 15, 2018, 13:35	3	Unknown	D5, D6
Jan 17, 2018, 20:05	2	Unknown	D4, L5
Jan 20, 2018, 15:05	3	Unknown	D4, L5
Jan 25, 2018, 7:05	1	Unknown	D5, D6
Jan 26, 2018, 11:35	0	Unknown	D3, D4, D6, L4, L5, C7
Jan 27, 2018, 12:35	3	Unknown	D4, D5, L5
Jan 28, 2018, 13:05	1	Unknown	D5, SA

Jan 29, 2018, 6:05	1	Unknown	D4, L5
Jan 29, 2018, 6:35	1	Unknown	D5, D6

Source Attribution and Peak Properties at H1

Source apportionment and the average emission rate of each organosilicon species detected in H1 are presented in Table S7. During summer, D3 and D4 emissions are dominated by continuous sources whereas D5 has similar contributions from episodic and continuous sources. Outdoor contributions to D3 and D4 are significant in summer, but are much smaller in winter. D6 is dominated by episodic emissions. D3 and SA emissions have approximately equal contributions from episodic and continuous sources. Both D5 and D6 siloxanes are dominated by episodic releases. D4 is dominated by continuous sources in both seasons. All these results are consistent with observations during H2W for the species reported in common between the two campaigns. Episodic emissions of D5 determined for H1S were generally an order of magnitude lower than during H1W and H2W. Episodic D3 emissions were consistent between H1S and H2W, but an order of magnitude lower in H1W. D4 and D6 emissions appeared to be consistent across both seasons at H1, with less variability than in H2. While the median episodic silyl acetate emission in H1W was similar to that determined in H2W, the mean and 90th percentile episodic emission values were 2.5× and 5.3× lower, respectively.

Table S7. Source attribution (%), average total emission rates, and episodic mass emission statistics (mg) of detected cyclic siloxanes and organosilicon compounds in the living zone of H1S and H1W during the occupied periods[*,a].

Season	**Compound**	**Average Emission Rate (mg d^{-1})**	**Source Attribution**			**Episodic Emissions**			
			Episodic	Continuous	Outdoor	Mean (mg)	Median (mg)	90th (mg)	No. Peaks
H1S	**D3**	3.8	24%	64%	12%	2.8	2.6	5.6	18
	**D4**	5.0	27%	51%	22%	3.1	2.0	6.7	23
	**D5**	49	48%	48%	4%	39	14	111	34
	**D6**	8.2	68%	31%	1%	6.3	4.5	15	10
H1W	**D3**	0.5	48%	48%	4%	0.23	0.20	0.41	25
	**D4**	11	22%	78%	1%	3.7	1.6	8.2	23
	**D5**	750	92%	7%	0.5%	720	100	2300	21
	**D6**	4.0	80%	14%	6%	5.2	5.2	8.8	9
	**SA**	1.1	51%	45%	4%	0.61	0.66	0.87	12

[*] Mean, median, and 90th percentile of the mass emitted during episodic events; No. Peaks = number of peaks analyzed.

[a] The occupied period refers to these dates spanning 48 (S) and 30 (W) days, respectively: Aug 11-Sept 27 (S) and Jan 31-Mar 01 (W).

Source Attribution of D3 and D4 in 'Thanksgiving' Experiments at HOMEChem

Figure S3 shows D5 siloxane plotted on the top panel and D3, D4, and D6 plotted on the bottom, on the left for the first 'Thanksgiving' day, and on the right side for the second. While D5 and D6 siloxanes show peaks occurring at the times that the cooks entered in the morning and the guests entered in the afternoon, D3 and D4 siloxanes gradually increase throughout the day with peak concentrations in the afternoon. Seeing that D3/D4 and D5/D6 were not driven by the same factors during these experiments, D3 and D4 were plotted with room temperature in Figure S4. The elevated concentrations of D5 and D6 are likely due to higher occupancy and potential diversity in personal care product usage as compared to other experimental days; however, as records were not kept of what products were used, the authors cannot comment on this point further.

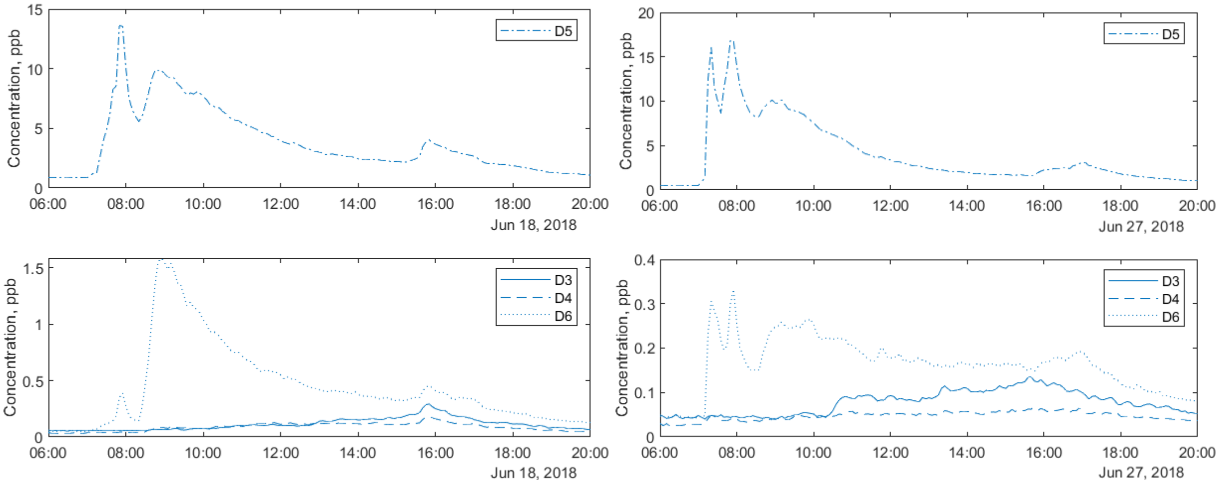

Figure S3. Time series of cyclic VMS during two HOMEChem 'Thanksgiving' experiments. D5 siloxane is presented in the top panels ((a) and (c)) while D3, D4, and D6 siloxanes are presented in the bottom panels ((b) and (d)).

Based on results reported in Table S3, on most HOMEChem experimental days, the four cVMS species varied with occupancy and personal care product usage, with D5 being the most abundant. However, on the days of the simulated Thanksgiving meal, D3 and D4 siloxanes behaved differently from D5 and D6 siloxanes. (See Figure S3.) Figure S4 shows the time series of D3 and D4 siloxane concentrations along with the time-varying room temperature during the two HOMEChem Thanksgiving experiments (a and b). For these experiments, 4-6 people entered the house at 8:30 AM to cook Thanksgiving dinner and an additional group of ~10 people entered at about 15:30 to eat the meal. After cleaning, all occupants left at 17:00. In the first experiment (left frame of Figure S4), the D3 concentration profile follows the room temperature profile closely, with little evident influence of occupant activity. Occupancy appears to have had more of an effect on D4, as it increases slightly both when the cooks and when the guests enter. However, the D4 concentration profile also appears to vary with room temperature. During the second HOMEChem Thanksgiving experiment (right frame), D3 once again follows a trend similar to that of the temperature profile, though the relationship is weaker than during the first Thanksgiving experiment. The D4 concentration profile shows a weak relationship to both room temperature and occupancy. From these results, it appears that both D3 and D4 siloxanes have sources other than personal care products; however, the specific source(s) are unknown.

A working hypothesis is that D3, a known PDMS thermal degradation product [14], was emitted during intensive oven use in the Thanksgiving experiments. We have previously reported that larger, low-volatility siloxanes were emitted from oven use on these experimental days [15]. An alternative explanation is also feasible: that the change in room temperature because of oven use caused re-emission of D3 and D4 previously sorbed in surface reservoirs. A possible primary source of the D3 and D4 emissions is PDMS- or other silicone-containing products, such as adhesives, lubricants, or heat-transfer compounds, located in or near the oven. Perhaps both hypotheses are correct: one may be driving D3 emissions while the other is driving D4 emissions, or both the oven usage and the room temperature are influencing emissions of both compounds. During the three observational campaigns in ordinarily occupied houses, oven use was never sufficient in intensity or duration to separately explore these hypotheses.

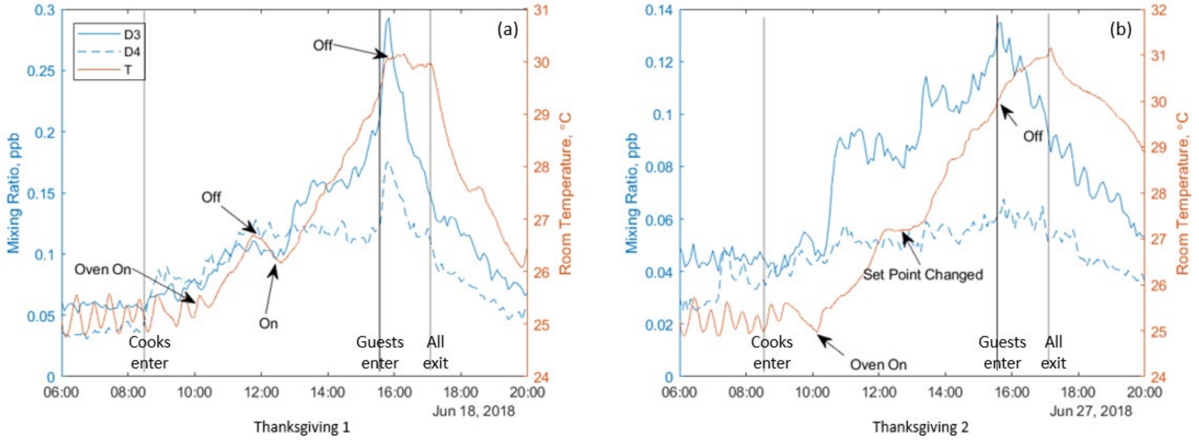

Figure S4. Time series of D3 and D4 concentrations along with room temperature during HOMEChem Thanksgiving experiments 1 (a) and 2 (b). Each panel has three vertical lines: the first vertical line indicates when the "cooks" enter, the second indicates when the "guests" enter, and final line indicates when all occupants exit the house. The arrows indicate the times at which oven settings were changed.

The mass of D3 and D4 effectively emitted during each Thanksgiving experiment was determined using eq S1, and is presented in Figure S5. The "background-subtracted" emissions, or the amount of a compound emitted that is not attributable to background sources, was estimated from eq S3. The amount emitted from personal care products was also estimated using eq S3 but integrated only over the peaks attributed to cooks or guests entering the test house rather than over the entire experiment. The remaining amount was assigned to an "unattributed" source category. Over the course of the first Thanksgiving experiment, about 1.3 mg of D3 and 1.4 mg of D4 were emitted, and over the course of the second Thanksgiving experiment, about 0.9 mg of D3 and 0.7 mg of D4 were emitted. On average, only 10% of D3 emissions were attributed to personal care products and 39% were attributed to background sources, leaving half of the estimated emissions unattributed to a known source category, as shown in Table S8. For D4, 25% emissions were attributed to personal care products and 31% to background sources, leaving just under half of the estimated emissions unattributed, also in Table S8. In summary, on both Thanksgiving days, D3 and D4 siloxane emissions were dominated by what is hypothesized to be oven use or a temperature-driven source category, with smaller, yet still substantial emissions from an unidentified background source.

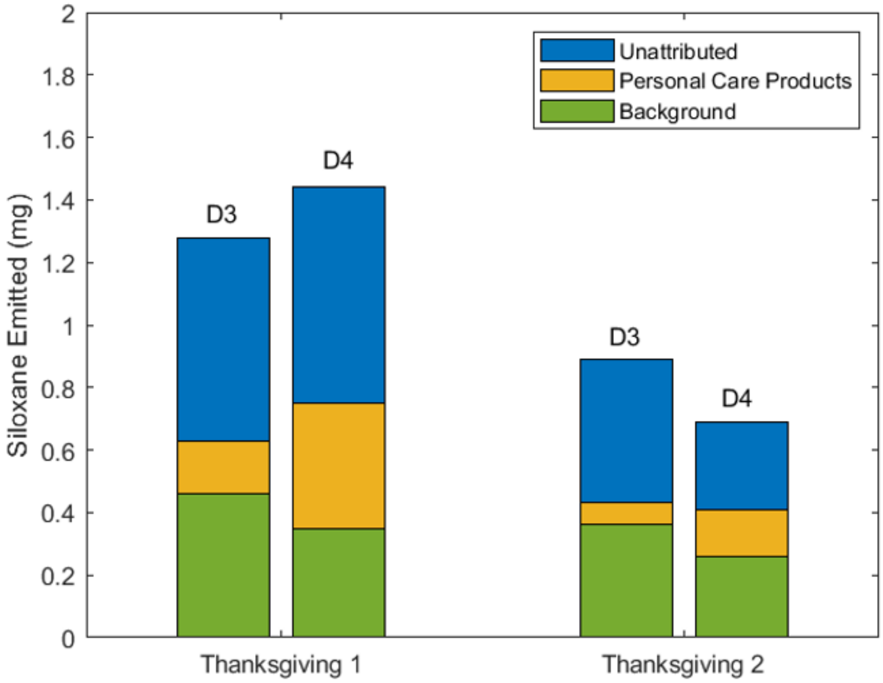

Figure S5. Amount of D3 and D4 emitted during each Thanksgiving event in HOMEChem, apportioned by source category. Green represents estimated continuous emissions resulting from stationary indoor sources, yellow indicates estimated emissions from personal care products, and blue shows the remaining unattributed emissions.

Table S8. Source apportionment of D3 and D4 emissions during HOMEChem 'Thanksgiving' experiments.

**Source**	**Thanksgiving 1**		**Thanksgiving 2**	
	**D3**	**D4**	**D3**	**D4**
Personal Care Product	13%	28%	7%	22%
Background	36%	24%	41%	38%
Unattributed	51%	48%	52%	40%
**Total Emissions**	1.3 mg	1.4 mg	0.9 mg	0.7 mg

References

1. Liu, Y.; Misztal, P. K.; Xiong, J.; Tian, Y.; Arata, C.; Weber, R. J.; Nazaroff, W. W.; Goldstein, A. H. Characterizing sources and emissions of volatile organic compounds in a northern California residence using space- and time-resolved measurements. *Indoor Air* **2019**, *29*, 630–644.
2. Greve, K.; Nielsen, E.; Ladefoged, O. (Eds.). *Siloxanes (D3, D4, D5, D6, HMDS). Evaluation of health hazards and proposal of a health-based quality criterion for ambient air.* Environmental Project No. 1531. Denmark: The Danish Environmental Protection Agency. 2014. Accessed 13 September 2021 at https://www2.mst.dk/Udgiv/publications/2014/01/978-87-93026-85-8.pdf.
3. Kochetkov, A.; Smith, J. S.; Ravikrishna, R.; Valsaraj, K. T.; Thibodeaux, L. J. Air-Water Partition Constants for Volatile Methyl Siloxanes. *Environ. Toxicol. Chem.* **2001**, *20*, 2184–2188.
4. Dodecamethylpentasiloxane. PubChem. National Library of Medicine. Accessed October 31, 2021, at https://pubchem.ncbi.nlm.nih.gov/compound/Dodecamethylpentasiloxane.
5. Raj, P. S. Amended Safety Assessment of Dimethicone, Methicone, and Substituted-Methicone Polymers as Used in Cosmetics. Expert Panel for Cosmetic Ingredient Safety. Revised Tentative Amended Report for Public Comment, January 12, 2021. Accessed October 31, 2021, at https://www.cir-safety.org/sites/default/files/methic122020revTAR.pdf.
6. Silyl acetate. ChemSpider. Royal Society of Chemistry. Accessed October 31, 2021, at http://www.chemspider.com/Chemical-Structure.4416754.html.
7. Xu, S.; Kozerski, G.; Mackay, D. Critical Review and Interpretation of Environmental Data for Volatile Methylsiloxanes: Partition Properties. *Environ. Sci. Technol.* **2014**, *48*, 11748–11759.
8. Weschler, C. J.; Nazaroff, W. W. Semivolatile organic compounds in indoor environments. *Atmospheric Environment* **2008**, 42, 9018–9040.
9. Fromme, H.; Debiak, M.; Sagunski, H.; Röhl, C.; Kraft, M.; Kolossa-Gehring, M. The German approach to regulate indoor air contaminants. *International Journal of Hygiene and Environmental Health* **2019**, *222* (3), 347–354.
10. Kristensen, K.; Lunderberg, D. M.; Liu, Y.; Misztal, P. K.; Tian, Y.; Arata, C.; Nazaroff, W. W.; Goldstein, A. H. Sources and dynamics of semivolatile organic compounds in a single-family residence in northern California. *Indoor Air* **2019**, *29*, 645–655.
11. Arata, C.; Misztal, P. K.; Tian, Y.; Lunderberg, D. M.; Kristensen, K.; Novoselac, A.; Vance, M. E.; Farmer, D. K.; Nazaroff, W. W.; Goldstein, A. H. Volatile organic compound emissions during HOMEChem. *Indoor Air* **2021,** *31*, 2099–2117.
12. Lunderberg, D. M.; Misztal, P. K.; Liu, Y.; Arata, C.; Tian, Y.; Kristensen, K.; Weber, R. J.; Nazaroff, W. W.; Goldstein, A. H. High-Resolution Exposure Assessment for Volatile Organic Compounds in Two California Residences. *Environ. Sci. Technol.* **2021**, *55*, 6740-6751.
13. Wang, C.; Collins, D. B.; Arata, C.; Goldstein, A. H.; Mattila, J. M.; Farmer, D. K.; Ampollini, L.; DeCarlo, P. F.; Novoselac, A.; Vance, M. E.; Nazaroff, W. W.; Abbatt, J. P. D. Surface reservoirs dominate dynamic gas-surface partitioning of many indoor air constituents. *Sci. Adv.* **2020**, *6*, eaay8973.
14. Camino, G.; Lomakin, S. M.; Lazzari, M. Polydimethylsiloxane thermal degradation. Part 1. Kinetic aspects. *Polymer* **2001**, *42*, 2395–2402.

15. Katz, E. F.; Lunderberg, D. M.; Brown, W. L.; Day, D. A.; Jimenez, J. L.; Nazaroff, W. W.; Goldstein, A. H.; DeCarlo, P. F. Large Emissions of Low-Volatility Siloxanes during Residential Oven Use. *Environ. Sci. Technol. Lett.* **2021**, *8*, 519–524.
